# Lymphatic vessel density and prognosis in cutaneous melanoma

**DOI:** 10.1038/sj.bjc.6602122

**Published:** 2004-08-17

**Authors:** O Straume, L A Akslen

**Affiliations:** 1 Department of Pathology, The Gade Institute, Haukeland University Hospital, N-5021 Bergen, Norway

**Sir**,

Recent studies have focused on the possible prognostic importance of lymphatic vessel density (LVD) in malignant melanoma, and we have read the paper by Shields *et al* (2004) with great interest. Based on their data, the authors suggest that the assessment of lymphatic vessel density (LVD) might give prognostic information in cutaneous melanoma. As they point out, some conclusions of their study might, in part, be in discordance with our own report (Straume *et al*, 2003), and we would therefore like to discuss some aspects of their work.

So far, relatively few survival studies on lymphangiogenesis have been published. In 2001, Birner *et al* (2001) presented a study on cervical cancer, and later our group published the first survival study of melanomas, based on 175 cases of aggressive vertical growth phase melanoma of the nodular type (median thickness 3.6 mm) (Straume *et al*, 2003). The results of both these studies indicate that increased LVD is associated with improved patient survival. More recently, however, Valencak *et al* (2004) published a prognostic study of 120 melanoma patients showing a weak association between increased LVD and reduced survival, although in univariate analysis only. In a study by Dadras *et al* (2003), based on the selection of 19 nonmetastatic melanoma patients and 18 patients with early lymph node metastasis, intratumour LVD was found to be increased in the metastatic group. In the study of Shields *et al*, including 21 melanoma cases (eight nonmetastatic, mean thickness 2.3 mm, and 11 metastatic, mean thickness 3.4 mm), LVD was also higher in tumours that developed metastases. Interestingly, LVD tended to decrease with increasing tumour thickness (their Table 2). No follow-up information (time to events) was presented. Whereas these three studies (Dadras *et al*, 2003; Shields *et al*, 2004; Valencak *et al*, 2004) seem to imply that increased LVD is associated with tumour progression and poor prognosis, the contrary was found in our own study (Straume *et al*, 2003). What could explain this difference?

Several explanations might be considered. We agree with Shields *et al* that the ‘hot spot’ technique, originally described by Weidner *et al* (1991) for the estimation of angiogenesis, might be more applicable for blood microvascular capillaries, and this approach is more subjective. The authors discuss whether some of the differences in our results could be attributed to this fact. Although our two studies showed comparable values of mean epi/peri-tumoral LVD (10.0 *vs* 14.3 mm^−2^), we wanted to address this issue and selected 19 lethal and 21 nonlethal melanoma cases from our series (Straume *et al*, 2003), and tumours were matched for thickness. Lymphatic vessels were counted along the complete tumour border in consecutive high power fields (HPF, × 400), and vessels more than 1 HPF away from the invasive border were not counted. We found that the median absolute LVD (LVD-ABS) was 5.4 vessels mm^−2^ compared with 12.5 mm^−2^ by the ‘hot spot’ technique (LVD-HS) (7.0 in nonlethal cases, 3.4 in lethal cases), and LVD-ABS and LVD-HS were highly correlated (linear regression *R*=0.74, *P*<0.001 for continuous variables, and Pearson *χ*^2^ *R*=0.7, *P*<0.001 when categorised between lymphatic vessel rich or poor cases). Most importantly, the analysis of both overall survival (melanoma deaths only) and recurrence-free survival showed the same association for both LVD-ABS and LVD-HS, that is, improved survival by increased LVD-ABS (log rank *P*=0.005, *n*=40) and increased LVD-HS (log rank *P*<0.0001, *n*=169). These data indicate that the difference in results cannot be explained by the difference in counting technique only. Still, we agree that LVD-ABS might be more appropriate and more easily standardised than LVD-HS, as suggested by Shields.

In the study by Dadras *et al* (2003), 18 metastatic and 19 nonmetastatic melanomas were included (mean thickness 2.5 mm, 68% Clark 4–5; mainly (76%) superficial spreading melanomas). The median peritumoral LVD (12.8 mm^−2^) was almost identical with our own findings (12.5 mm^−2^), suggesting that both methods (Dadras: computer-assisted morphometric analysis; Straume: counting in ‘hot spot’ areas a.m. Weidner) gave similar results. Even if low reproducibility is considered an important problem of vessel counting (Hlatky *et al*, 2002), this method is frequently used in translational angiogenesis research; other methods, including morphometry, have not proved to be superior.

The study of Valencak *et al* (2004) included 120 cases of cutaneous melanoma with follow-up information (median thickness not given; 18% of patients were Clark 0–2, 45% were Clark 4–5; in comparison, 90% were Clark 4–5 in our own study). By staining for podoplanin, and using the ‘hot spot’ method of counting, the mean LVD was 11.3 per field (field size not given), and this is probably considerably higher than in the other studies. Increased LVD was associated with decreased survival, but the difference was only modest and did not persist in multivariate analysis, in contrast to what we found (Straume *et al*, 2003). The use of different markers could be one explanation for the findings, although LVD by LYVE-1 and podoplanin staining was significantly correlated in our previous study. Also, the sensitivity and specificity of the LYVE-1 antibody were evaluated in a recent study by Akishima *et al* (2004).

It should be pointed out that our series (Straume *et al*, 2003) consisted of thicker and more advanced primary melanomas than the other series, consisting of vertical growth phase melanomas only. This could be an important explanation why the relationship between LVD and progression/prognosis appears to be different. Interestingly, we found a significant association between increased LVD and lymphocytic infiltration, which is a favourable prognostic factor for cutaneous melanomas, and a similar relationship with lymphatic vascular area was found by Dadras *et al* (2003). This points to a relationship between lymphatic vessels and an antitumour immune response.

The studies of Dadras *et al* and Shields *et al* have included a low number of pre-selected cases and might not be classified as prognostic factor studies according to the criteria of Simon and Altman (1994). For instance, Dadras *et al* did not present prognostic information on LVD, but found that relative lymphatic vessel area was a prognostic factor, although their cut-points appear to be selected on the basis of a relationship with a strong predictor of outcome, that is, lymph node metastases (their Figure 6A). The ‘prognostic’ information from these studies should therefore be interpreted with great care. A summary of studies of clinical end points according to lymphatic markers in human cancers has recently been given by Padera *et al* (2003).

The results of animal studies have suggested that increased lymphangiogenesis may be important for the frequency of lymphatic metastasis, and the recent identification of novel lymphangiogenic factors, like VEGF-C and VEGF-D, has increased the focus on lymphangiogenesis considerably (Kaipainen *et al*, 1995; Achen *et al*, 1998; Skobe *et al*, 2001; Stacker *et al*, 2001). Still, the evidence provided in the literature is not sufficient to conclude that active lymphangiogenesis play an important role in human cancer. As for the difference between studies on cutaneous melanoma, the balance between LVD as a marker of lymphatic spread, and its association with lymphocytic infiltration and antitumour immune activity, might differ between early (thinner) and late stage (thicker) melanomas. Also, the relative contribution of lymphogenic and hematogenic metastases may be different between these tumour subgroups. We feel that more survival studies, following the criteria of Simon and Altman (1994), are needed before the estimation of lymphangiogenesis is applicable to clinical decision making and prognostication in melanoma patients.
